# Cognitive decline related to chronic kidney disease as an exclusion factor from kidney transplantation: results from an international survey

**DOI:** 10.1093/ckj/sfae114

**Published:** 2024-04-13

**Authors:** Michele Farisco, Inga A Blumblyte, Casper Franssen, Dorothea Nitsch, Irene Zecchino, Giovambattista Capasso, Gaye Hafez, COST CONNECT Action Collaborators, Giovambattista Capasso, Alexandre Andrade, Mustafa Arici, Maie Bachmann, Matthew Bailey, Michelangela Barbieri, Mickaël Bobot, Annette Bruchfeld, Inga Bumblyte, Antonello Calcutta, Giovanna Capolongo, Sol Carriazo, Michele Ceccarelli, Adrian Constantin Covic, Ananya De, Pilar Delgado, Nicole Endlich, Matthias Endres, Fabrizio Esposito, Michele Farisco, Quentin Faucher, Ana Carina Ferreira, Andreja Figurek, Denis Fouque, Casper Franssen, Ivo Fridolin, Sebastian Frische, Liliana Garneata, Loreto Gesualdo, Konstantinos Giannakou, Olivier Godefroy, Aleksandra Golenia, Dimitrios Goumenos, Agnė Gryguc, Eugenio Gutiérrez Jiménez, Gaye Hafez, Ewout Hoorn, Pedro Henrique Imenez Silva, Raafiah Izhar, Dearbhla Kelly, Shelli Kesler, Aleksandra Klimkowicz-Mrowiec, Samuel Knauss, Justina Kurganaite, Hélène Levassort, Sophie Liabeuf, Jolanta Malyszko, Laila-Yasmin Mani, Gianvito Martino, Ziad Massy, Christopher Mayer, Armida Mucci, Alma Mutevelic-Turkovic, Rikke Nielsen, Dorothea Nitsch, Alberto Ortiz, Vasileios Panagiotopoulos, Giuseppe Paolisso, Bojana Pejušković, Marion Pepin, Alessandra Perna, Andrea Perrottelli, Vesna Pešić, Pasquale Pezzella, Merita Rroji (Molla), Ivan Rychlík, Giorgos Sakkas, Mariadelina Simeoni, Maria José Soler Romeo, Goce Spasovski, Ana Starčević, Gioacchino Tedeschi, Francesco Trevisani, Robert Unwin, Evgueniy Vazelov, Carsten Alexander Wagner, Franca Wagner, Christoph Wanner, Andrzej Wiecek, Hong Xu, Miriam Zacchia, Lefteris Zacharia, Irene Zecchino, Carmine Zoccali, Francesco Mattace Raso, Karl Hans Endlich, Norberto Perico, Giuseppe Remuzzi, Francesco Trepiccione, Mark Okusa, Vincenzo Di Marzo, Peter Blankestijn, Kai-Uwe Eckardt, Maximilian Konig

**Affiliations:** Department of Public Health and Caring Sciences, Centre for Research Ethics and Bioethics, Uppsala University, Uppsala, Sweden; Biogem Molecular Biology and Genetics Research Institute, Ariano Irpino (AV), Italy; Department of Nephrology, Lithuanian University of Health Sciences, Kaunas, Lithuania; Department of Nephrology, University Medical Center Groningen, University of Groningen, Groningen, The Netherlands; UK Renal Registry, UK Kidney Association, Bristol, UK; Department of Non-communicable Disease Epidemiology, Faculty of Epidemiology and Population Health, London School of Hygiene and Tropical Medicine, London, UK; Department of Nephrology, Royal Free London NHS Foundation Trust, London, UK; Department of Science and Technology, University of Sannio, Benevento, Italy; Department of Public Health and Caring Sciences, Centre for Research Ethics and Bioethics, Uppsala University, Uppsala, Sweden; Department Translational Medical Science, University Campania Luigi Vanvitelli, Naples, Italy; Department of Pharmacology, Faculty of Pharmacy, Altinbas University, Istanbul, Turkey

**Keywords:** brain, chronic kidney disease, cognitive impairment, ethics, kidney transplantation

## Abstract

**Background and hypothesis:**

There seems to be a lack of consensus on the necessity and the modality of psychological and specifically cognitive assessment of candidates for kidney transplantation. Both points are often delegated to individual hospitals/centres, whereas international guidelines are inconsistent. We think it is essential to investigate professionals' opinions to advance towards a consistent clinical practice.

**Methods:**

This paper presents the results of an international survey among clinical professionals, mainly nephrologists from the CONNECT (Cognitive decline in Nephro-Neurology: European Cooperative Target) network and beyond (i.e. from personal contacts of CONNECT members). The survey investigated their opinions about the question of whether cognitive decline in patients with chronic kidney disease may affect their eligibility for kidney transplantation.

**Results:**

Our results show that most clinicians working with patients affected by chronic kidney disease think that cognitive decline may challenge their eligibility for transplantation despite data that suggest that, in some patients, cognitive problems improve after kidney transplantation.

**Conclusion:**

We conclude that three needs emerge as particularly pressing: defining agreed-on standards for a multifaceted and multifactorial assessment (i.e. including both clinical/medical and psychosocial factors) of candidates with chronic kidney disease to kidney transplantation; further investigating empirically the causal connection between chronic kidney disease and cognition; and further investigating empirically the possible partial reversibility of cognitive decline after kidney transplantation.

KEY LEARNING POINTS **What was known:**

- There is growing evidence about the impact of chronic kidney disease on cognition, specifically about the causal relationship between chronic kidney disease and cognitive impairment. This causal connection potentially raises the ethical issue of the patient's autonomy, particularly about their inclusion in kidney transplantation.

**This study adds:**

- This survey specifically investigated the professionals’ opinions on whether chronic kidney disease may be considered as an exclusion factor from kidney transplantation, providing empirical data to the ethical debate.

**Potential impact:**

- This survey may be a significant contribution to the reflection on the ethical implications of the connection between chronic kidney disease and cognitive impairment. Specifically, this survey provides valuable data from professionals that can inform the ethical reflection.

## INTRODUCTION

There is a growing interest in the association between kidney and brain function, which is the object of dedicated scientific and clinical investigation [1–9].

Patients on haemodialysis and peritoneal dialysis show a high burden of cognitive impairment [10, 11]. A cross-sectional survey found an association of chronic kidney disease (CKD) with an increased burden of cognitive impairment in the US general population [12]. In terms of underlying pathobiology, a potential mechanism for such an association is shared risk factors affecting vascular beds in both the brain and kidneys [13] that eventually have a negative impact on cognition [14, 15].

This high burden of cognitive impairment in patients with kidney disease (irrespective of kidney replacement therapy status) is highly relevant for clinical practice. One related topic that deserves a more in-depth analysis is the possible implications of cognitive impairment in these patients for some ethically and legally relevant notions, such as autonomy and self-determination, including their ability to provide valid informed consent. These notions play a crucial role in biomedical ethics and clinical regulation, and they are eventually necessary conditions for a clinical procedure (either therapeutic or experimental) to be compliant and reliable. Notably, cognitive impairment may affect both eligibility for kidney transplantation (KT) and post-KT outcomes, graft survival, adherence, quality of life, and health care costs.

The case of KT for patients with advanced CKD can be taken as an illustrative case study. Evaluations of specific cognitive capacities, including psychological, psychiatric, and psychosocial dimensions, are not consistently included in the pretransplant assessment of potential recipients. To illustrate, there is no reference to patients’ exclusion criteria, including possible psychological exclusion criteria, in the EAU Guidelines [16].

The recent Kidney Disease Improving Global Outcomes (KDIGO) Clinical Practice Guideline on the Evaluation and Management of Candidates for KT reserves important attention to the psychosocial evaluation of candidates [17]. Among other things, the KDIGO Guidelines recommend performing a psychological assessment on all the candidates, and not transplanting to patients who have an unstable psychiatric disorder that affects decision-making capacity (4.1; 4.2).

The rationale behind these recommendations is that ‘a comprehensive psychosocial assessment allows for identification of factors that may adversely impact the success of transplantation and for targeted interventions to be implemented, thereby enhancing the likelihood of a favourable outcome for the patient’ [17].

The KDIGO Guidelines are the most detailed and informative about the psychosocial assessment of potential candidates for KT, although not explicitly covering cognitive issues that are not the same as having psychiatric disease. Among other guidelines, the Canadian and the American Society of Transplantation indicate that mental illness alone is not a contraindication to transplantation and that patients with psychiatric or psychological disorders should be referred for treatment [18, 19], while the Australian guidelines are silent on evaluation and/or selection of candidates with a psychiatric or psychological disorder [20, 21], and New Zealand Guidelines suggest that patients with cognitive or neuropsychiatric deficits may not be appropriate transplant candidates if they do not have a caregiver to facilitate post-transplant medication adherence (https://www.kidney.health.nz/resources/files/picker/new-scheme-for-assessing-patients-v2-1.pdf). Finally, the European Best Practice Guidelines for Renal Transplantation does not contain explicit reference to the cognitive and psychosocial evaluation of candidates [22].

 In conclusion, there seems to be no consistency about how to deal with the cognitive assessment in general and the psychosocial assessment in particular of potential candidates for KT.

Against this background, the working group-5 of the COST Action CONNECT (Cognitive decline in Nephro-Neurology: European Cooperative Target) elaborated a survey to investigate the opinions and practices of relevant clinical professionals, both from within the CONNECT network and beyond. The major goals of this survey were to collect information about the actual procedures/protocols implemented by relevant professionals, to investigate their opinions about the question if and how cognitive decline associated with CKD may impact a patient’s eligibility for transplantation, and ultimately to inform the ethical reflection about cognitive decline possibly related to CKD as an exclusion factor from KT.

## MATERIALS AND METHODS

### Study population

Invitation to take part to the survey was sent to all CONNECT members (*n* = 84) and to other professionals from their personal contacts. Invited people were from different countries (*n* = 27) and disciplines (*n* = 8). Demographics of respondents are specified in the following subsections.

### Dissemination methods

An invitation letter, including a description of CONNECT, the rationale and goal of the survey, how to access it through a Google form, and specifying that participation in it was voluntary and anonymous, was sent by email. The first invitation was sent out on 4 July 2022; a first reminder was sent out on 1 August 2022; a third reminder on 19 August 2022; and a last reminder on 6 September after having postponed the original deadline (30 August) to 15 September. As the number of participants (152) in the first round of the survey was considered not sufficient, the survey was re-opened from 15 February to 29 February 2024.

### Questions

An international survey has been conducted involving different professionals working with CKD patients. The questionnaire included 11 closed questions. The first five were demographic:

(i) What is your age?
(ii) What is your gender?
(iii) Which country do you represent?
(iv) What is your speciality?
(v) How many years of professional experience do you have?

The other four were elaborated starting from the previously mentioned KDIGO Guidelines.

The KDIGO Guidelines analyse the issue of KT based on three key questions:

(i) Should all candidates have a psychosocial assessment?
(ii) Who should perform the psychosocial assessment?
(iii) How should the psychosocial assessment be performed?

Concerning point (i), as already mentioned, the KDIGO Guidelines recommend performing the psychosocial assessment for all candidates.

Concerning points (ii) and (iii), a face-to-face assessment is generally recommended. If not possible (e.g. in the case of a patient that is medically incapacitated and unable to reliably participate in an interview), the clinician might rely on collateral sources (e.g. family members, primary care physicians) for information to complete the psychosocial assessment.

Table 1 reports list of factors to assess through psychosocial evaluation according to the KDIGO Guidelines:

**Table 1:** Factors to assess in KT candidates according to KDIGO

a mental status examination
cognitive evaluation to ensure valid decision-making capacity and ability to provide informed consent for transplantation
understanding of the transplant process
motivation for transplantation
expectations of the outcomes (including graft/patient survival, symptom relief, and quality of life)
ability and willingness to form a collaborative relationship with the transplant team
past and current psychiatric/psychological disorders
past and current substance use (e.g. alcohol, tobacco, drugs)
past and current adherence to recommendations regarding medical treatment and lifestyle modifications
social history (e.g. education, occupation, financial resources, important relationships, living circumstances)
cultural factors relevant to chronic illness and transplantation
availability and stability of the social network as it pertains to meeting any caregiving needs of the patient

A balanced evaluation of these factors will eventually provide an answer about the feasibility of the transplantation. Importantly, the KDIGO Guidelines recommend that patients who are unable to engage independently in self-care activities have an identified support system in place before transplantation.

On the basis of the KDIGO Guidelines, we elaborated the following questions:

(vi) What is the annual number of KT patients treated at your centre?
(vii) Should all the patients with chronic kidney diseases have psychological assessment before being admitted to KT?
(viii) Who should perform the psychosocial assessment?
(ix) How should the psychosocial assessment be performed? Please select more than one.
(x) What inclusion criteria do you use in selecting patients for KT?’ Please select more than one option if relevant.
(xii) Can the cognitive decline become a contraindication to KT? (Reason).

### Ethics

We obtained consent from all respondents who were provided with information regarding the survey via the Google form.

This study was approved by the Altınbaş University Clinical Research Ethics Committee, Turkey (Approval Code 124, 24 June 2022). The study was conducted according to the principles established in the Declaration of Helsinki, the Council of Europe Convention on Human Rights and Biomedicine, and the requirements established in each COST CONNECT country legislation.

## RESULTS

The data were analysed using descriptive and analytical statistics calculated by Microsoft Excel 2022. Results ae presented in percentages.

A total of 346 participants from 27 countries answered the questions. Three participants from non-field specialties were excluded from the study. A total of 343 participants were included (Table 2). Of these, 313 participants were nephrologists (91%), and 30 participants (9%) were categorized as ‘others’ comprising eight geriatricians, eight neurologists, six psychiatrists, one internal medicine specialist, one clinical psychologist, one psychosomatic medicine specialist, one gastroenterologist, one general surgeon, one transplant surgeon, one toxicologist, and one paediatrician (Table 3). Age groups were divided as follows: under 30 years old (*n* = 14), 31–40 years old (*n* = 85), 41–50 years old (*n* = 110), 51–60 years old (*n* = 77), and 61 years and over (*n* = 57). Of them, 187 were men, 154 were women, and two participants preferred not to state their gender. Most of the respondents (71%) had >10 years of professional experience (Table 3). Almost half of the physicians (48%) who responded to the survey stated that the number of transplanted patients per year in their centres is >100 (Table 4).

**Table 2:** Responses per country

**Country**	Number	Percentage (%)
Albania	8	2.3
Belgium	6	1.7
Bosnia and Herzegovina	1	0.3
Canada	1	0.3
Cyprus	3	0.9
Czech Republic	1	0.3
Estonia	1	0.3
France	25	7.3
Germany	4	1.2
Greece	4	1.2
India	1	0.3
Italy	95	27.7
Jamaica	1	0.3
Latvia	1	0.3
Lithuania	18	5.2
Netherlands	20	5.8
Norway	1	0.3
Poland	9	2.6
Portugal	16	4.7
Republic of North Macedonia	3	0.9
Romania	6	1.7
Serbia	6	1.7
Spain	50	14.6
Sweden	9	2.6
Switzerland	4	1.2
Turkey	47	13.7
United Kingdom	2	0.6
Total	343	100.0

**Table 3:** Demographic data of the participants

Specialities	Number	Percentage (%)
Nephrology	313	91
Others	30	9
Age		
<30	14	4
31–40	85	25
41–50	110	32
51–60	77	22
>61	57	17
Gender		
Female	187	55
Male	154	45
Not prefer to say	2	1
Professional experience		
<5 years	40	12
5–10 years	61	18
>10 years	242	71

**Table 4:** Results of responses

	Number of responses (*n*)	Percentage (%)
Q6. What is the annual number of KT patients treated at your center?		
<50	85	25
Between 50 and 100	60	17
>100	158	46
None	40	12
Q7. Should all patients with chronic kidney diseases have a psychological assessment before being admitted to KT?		
yes	248	72
no	95	28
Q8. Who should perform the psychosocial assessment? (multiple choice)		
The clinician in charge of the patient	48	14
The clinician in collaboration with the patient's family	56	16
A specialized professional	151	44
A specialized professional in collaboration with the patient's family	161	47
Other (neurologist/neuropsychologist, geriatrician, social worker other than HCP)	4	1
Q9. How should the psychosocial assessment be performed? (multiple choice)		
Face-to-face assessment	318	93
Interviewing primary care physician	94	27
Interviewing family members	173	50
Interviewing patients' friends	27	8
Other (remote cognitive assessment, geriatric, or neurologist assessment, questionnaires)	8	2
Q10. What inclusion criteria do you use in selecting patients for KT? (multiple choice)		
Physiological criteria	228	66
Psychiatric criteria	203	59
Psychosocial criteria	224	65
Other (condition of the patient such as malignancy, physical condition, life expectancy)	16	5
Q11. Can cognitive decline become a contraindication of KT?		
yes	213	62
no	130	38

Most of the respondents (72%) expressed ‘yes’ for Q7 asking should all patients with CKD should have a psychosocial assessment before being wait-listed for KT. About half of the respondents reported a specialized professional (44%) and/or a specialized professional in collaboration with the patient's family (47%) should perform a psychosocial assessment, respectively. Almost all the respondents (93%) reported that face-to-face assessment is required. The other responses for the psychosocial assessment method were interviewing family members (50%), interviewing primary care physicians (27%), and interviewing patients' friends (8%), respectively (Table 4).

Among all participants, most reported the inclusion criteria used in selecting patients for KT as physiological criteria (66%), psychosocial criteria (65%), and psychiatric criteria (59%), respectively. More than half of all respondents (62%) reported that cognitive decline may be a contraindication for KT (Table 4). There were 167 respondents who considered cognitive impairment as a potential contraindication for KT and who gave some reasons to the open-ended part of the question why cognitive impairment might be an exclusion factor in KT (Table 5). The main reason quoted were concerns about patient compliance during the post-transplantation treatment period (*n* = 102). Other listed reasons were a patient's bad prognosis or severe impairment (*n* = 45), concerns about the patient's capacity in decision-making and self-care (*n* = 27), presence of dementia (*n* = 18), absence of any socio-family support *(n* = 18), need for a psychosocial assessment (*n* = 12), social concerns (utilitarian view, scarce source of organs) (*n* = 11), and concerns about post-transplantation complication risks such as infection (*n* = 9) (Table 5).

**Table 5:** Classification of the reasons given by those who said ‘yes’ to question Q11

Category	Classification of the reasons given by those who said ‘yes’ to question Q11 (multiple reasons)	Number (*n*)
Category 1	Concerns about post-transplantation treatment patient's compliance	102
Category 2	If there is bad prognosis or severe impairment	45
Category 3	Concerns about patient capacity in decision-making and self-care	27
Category 4	Only in case of dementia	18
Category 5	If there is no socio-family support	18
Category 6	Depending on the psychological assessment	12
Category 7	Social concerns (utilitarian view, scarce source of organs)	11
Category 8	Concerns about post-transplantation complication risks such as infection	9

## DISCUSSION

From this survey, it emerges that most professionals are in line with KDIGO Guidelines about the patients’ assessment and the inclusion criteria for KT. In fact, most participants declared that transplantation candidates should be subject to psychosocial assessment before KT. Significantly, most respondents think that the cognitive decline associated with kidney disease may become a contraindication to KT.

In short, three main needs emerge from this survey: the need to perform a multifaceted and multifactorial assessment (i.e. including both clinical/medical and psychosocial factors) of CKD candidates to KT; the need to further investigate empirically the causal connection between CKD and cognition; and the need to further investigate empirically the possible partial reversibility of cognitive decline after KT.

**Figure 1:**
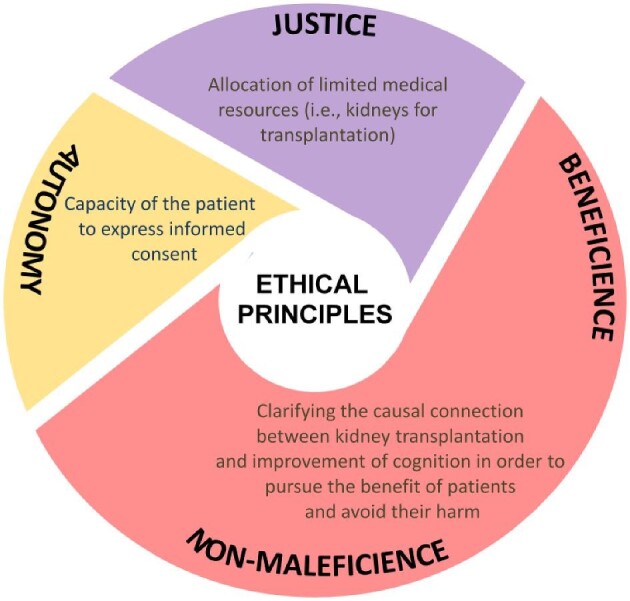
Ethical principles and emerging issues related to KT in CKD patients.

CKD raises a number of ethical issues that can be explored within the framework of traditional medical ethics, with specific reference to the principles of autonomy (i.e. self-determination), beneficence (i.e. maximizing good), non-maleficence (i.e. not causing harm), and justice (i.e. providing what is due) [23]. For instance, an ethical assessment of CKD within this ethical framework has been developed with specific focus on appropriate initiation or withdrawal of dialysis [24]. To illustrate the ethical implications of this survey, it is possible to develop a contextual model building on the analysis by Davison and Holley (Fig. 1).

In the context of KT, resources (i.e. organ availability) are limited, so that the issue of their just allocation arises. An additional open question is whether there is a causal connection between CKD and cognitive impairment, which is increasingly revealed by recent research [25, 26], and whether this is reversible or worsened by KT. The main ethical challenge to the principle of justice is the risk of using a limited resource for a CKD patient who will possibly have limited or no benefit from it if dementia worsens. This challenge may be assessed within different ethical frameworks. From a utilitarian/consequentialist point of view, this risk would suggest giving priority to those CKD patients with a better overall condition. Yet this conclusion is not straightforward. First, utilitarianism/consequentialism is only one possible ethical model: other frameworks are possible (e.g. virtue ethics, personalism, deontologism, etc.), with different conclusions on the point. Second, within the same utilitarian/consequentialist approach, there is an additional factor to take into account that challenges the conclusion mentioned previously. Providing a new kidney to a CKD patient could improve his/her cognitive profile, as revealed by recent data [27], which would eventually be an improvement of overall utility as dictated by the utilitarian/consequentialist model. How do we disentangle this challenging puzzle? Two hypotheses seem reasonable: (i) further empirical exploration about the connection between restored kidney functionality and cognitive performance in transplanted CKD patients, possibly taking into account other relevant clinical and systemic variables such as adequacy of haemodialysis, blood pressure, heart contraction during haemodialysis, concurrent medications, etc.; and (ii) a case-by-case assessment that takes into account additional extra-medical factors (e.g. those indicated in our survey, such as post-transplantation treatment patient compliance, the level of psychological impairment, availability of family support, prognosis) that may eventually influence the effectiveness of KT in CKD patients. In the latter case, a personalized multifactorial or multi-dimensional decision (i.e. considering several individual medical and psychosocial factors) would be ethically recommended.

Concerning the principles of beneficence and non-maleficence, KT for CKD patients raises the ethical dilemma whether transplanting is the right choice to serve the patients’ best interests. If the improvement of overall cognition status as a result of KT is confirmed, then performing the transplantation would be compliant with the ethical requirements to make the patients well and to not cause them harm, where the harm may be in the negative form of not providing a benefit (It may be argued that even if cognition would not improve, the overall quality of life of the CKD patient may still improve. For instance, he would be free of dialysis with its associated side-effects and consequences on the patient's daily life. The question is then raised as to whether the relative improvement of the patient's quality of life is sufficient to justify a particularly invasive procedure such as KT). Yet, if no sufficient data in support of the causal connection between KT and improvement of cognition are available, then performing transplantation in CKD patients may be against the ethical need for pursuing their benefit and avoiding their harm. For instance, the patients, their family, and possibly the donors may be subjected to an undue and not justified invasive clinical procedure. In conclusion, in the case of the principles of beneficence and non-maleficence, more empirical and clinical data are also required to make an ethically sound decision.

Finally, regarding the principle of autonomy, KT for CKD patients raises what is probably the most challenging ethical issue: how to assess and facilitate the patient's capacity to express his/her informed consent. In fact, being cognitively impaired, the patient may be unable to fully understand the medical information provided, which is a necessary requirement for expressing a valid informed consent.

In fact, according to the most accepted view in biomedical ethics, there are three necessary requirements for informed consent to be valid [28]: (i) disclosure, (ii) capacity, and (iii) voluntariness.

Disclosure requires that the clinician (i) gives the patient all the needed information for an autonomous decision and (ii) checks the adequate understanding of the information by the patient. Thus, the clinician must provide adequate and complete information to the patient and ensure that she/he has a sufficient understanding of provided information.

Capacity entails that the patient is not only able to understand the information provided by the clinician, but also to make a reasonable evaluation/anticipation of the potential consequences of the decision that she/he will make based on that information.

Voluntariness refers to the patient's right to decide without undue coercion or influence. In short, the necessary conditions for a patient’s decision expressed through informed consent to be autonomous are the following: (i) understanding the relevant information concerning the treatment and related risks and benefits, (ii) appreciating different therapeutic methods and related consequences, (iii) reasoning about different options, and (iv) communicating a personal choice [29, 30].

The cognitive decline potentially associated with CKD may impact these conditions for a valid informed consent. Therefore, it is important to have clear and consistent standards for assessing this possible impact.

Our survey provides relevant data in this direction. Despite the lack of a consistent international regulation on the topic, most of our respondents declared to be compliant with KDIGO recommendations to perform the psychosocial assessment for all the candidates to KT, as well as with the recommendation to perform this assessment face-to-face. Also, most think that a specialized professional in collaboration with the patient's family should perform this assessment (While it would be instrumental to the respect of the patient's autonomy, the involvement of his family may raise the risk of not respecting the patient's privacy if he does not want a family member to be present. This is an example of potential conflicts among different ethical principles deserving further specific analysis.). Therefore, clinical practice appears generally consistent with KDIGO recommendations concerning the psychosocial assessment of potential candidates to KT.

Concerning cognitive impairment specifically, there are different tests available to screen it, but there is the potential problem arising from the fact that these tests were introduced for assessing cognitive impairment in patients with Alzheimer's and may be not well suited for measuring cognitive impairment in CKD. One tool that is gaining traction in measuring cognition in CKD patients is the Montreal Cognitive Assessment (MoCA). Significantly, a recent study showed that pre-KT cognitive function, assessed through the MoCA, is a poor predictor of post-KT cognitive status [4], suggesting that pre-KT cognitive status should not be taken as the unique factor informing decision about KT. Another problem with the MoCA is that it is not validated for multi-ethnic populations and, in many settings, patients with advanced CKD come from a multi-ethnic background.

Concerning the specific issue of patient's selection for KT, psychosocial, and physiological criteria are almost equally taken into account by respondents. Interestingly, for most of them cognitive decline associated with CKD can become a contraindication to KT. Specifically, respondents tend to exclude cognitively impaired patients from transplantation if some additional factors are present (see Table 5), namely if the patient's compliance with post-transplantation treatment is compromised, if there is a bad prognosis or a severe impairment, and if the cognitive decline is so serious to raise concerns about the patient's capacity in decision-making and self-care. Therefore, the cognitive and psychosocial evaluation of candidates to KT is considered a crucial component for the eligibility of candidates to KT. This point is consistent with a recent Delphi Study, which also outlined the necessity of specific ethics guidelines about the cognitive assessment of candidates for KT [31].

## CONCLUSION

Consistent standards about the connection between CKD and cognitive decline are needed. Specifically, how to make an ethically sound decision about KT in CKD patients is a challenge. We conducted a survey among clinicians that provides relevant data in this direction. Three points emerge in particular: the need to perform a multifaceted and multifactorial assessment (i.e. including both clinical/medical and psychosocial factors) of CKD candidates to KT; the need to further investigate empirically the causal connection between CKD and cognition; and the need to further investigate empirically the possible partial reversibility of cognitive decline after KT.

## Data Availability

The raw data that support the findings of this study are available in OSF with the identifier https://osf.io/eh5ck/DOI 10.17605/OSF.IO/EH5CK.

## APPENDIX

The CONNECT collaborators are: Giovambattista Capasso, Alexandre Andrade, Mustafa Arici, Maie Bachmann, Matthew Bailey, Michelangela Barbieri, Mickaël Bobot, Annette Bruchfeld, Inga Bumblyte, Antonello Calcutta, Giovanna Capolongo, Sol Carriazo, Michele Ceccarelli, Adrian Constantin Covic, Ananya De, Pilar Delgado, Nicole Endlich, Matthias Endres, Fabrizio Esposito, Michele Farisco, Quentin Faucher, Ana Carina Ferreira, Andreja Figurek, Denis Fouque, Casper Franssen, Ivo Fridolin, Sebastian Frische, Liliana Garneata, Loreto Gesualdo, Konstantinos Giannakou, Olivier Godefroy, Aleksandra Golenia, Dimitrios Goumenos, Agnė Gryguc, Eugenio Gutiérrez Jiménez, Gaye Hafez, Ewout Hoorn, Pedro Henrique Imenez Silva, Raafiah Izhar, Dearbhla Kelly, Shelli Kesler, Aleksandra Klimkowicz-Mrowiec, Samuel Knauss, Justina Kurganaite, Hélène Levassort, Sophie Liabeuf, Jolanta Malyszko, Laila-Yasmin Mani, Gianvito Martino, Ziad Massy, Christopher Mayer, Armida Mucci, Alma Mutevelic-Turkovic, Rikke Nielsen, Dorothea Nitsch, Alberto Ortiz, Vasileios Panagiotopoulos, Giuseppe Paolisso, Bojana Pejušković, Marion Pepin, Alessandra Perna, Andrea Perrottelli, Vesna Pešić, Pasquale Pezzella, Merita Rroji (Molla), Ivan Rychlík, Giorgos Sakkas, Mariadelina Simeoni, Maria José Soler Romeo, Goce Spasovski, Ana Starčević, Gioacchino Tedeschi, Francesco Trevisani, Robert Unwin, Evgueniy Vazelov, Carsten Alexander Wagner, Franca Wagner, Christoph Wanner, Andrzej Wiecek, Hong Xu, Miriam Zacchia, Lefteris Zacharia, Irene Zecchino, Carmine Zoccali, Francesco Mattace Raso, Karl Hans Endlich, Norberto Perico, Giuseppe Remuzzi, Francesco Trepiccione, Mark Okusa, Vincenzo Di Marzo, Peter Blankestijn, Kai-Uwe Eckardt, and Maximilian Konig.

## References

[1] Zachciał J, Uchmanowicz I, Czapla M et al. The association between psychosocial and age-related factors with adherence to immunosuppressive therapies after renal transplantation. J Clin Med 2022;11:2386. 10.3390/jcm11092386 35566514 PMC9105664

[2] De Pasquale C, Pistorio ML, Veroux M et al. Psychological and psychopathological aspects of kidney transplantation: a systematic review. Front Psychiatry 2020;11. 10.3389/fpsyt.2020.00106 PMC7066324 32194453

[3] Lullmann O, Conrad AL, Steinbach EJ et al. Neurocognitive deficits may not resolve following pediatric kidney transplantation. Pediatr Transplant 2023;27:e14505. 10.1111/petr.14505 36932049 PMC11001201

[4] Gupta A, Montgomery RN, Young K et al. Pre-transplant cognitive screening is a poor predictor of post-transplant cognitive status. Clin Transplant 2022;36:e14798. 10.1111/ctr.14798 35989467 PMC10691449

[5] Vinke JSJ, Ziengs AL, Buunk AM et al. Iron deficiency and cognitive functioning in kidney transplant recipients: findings of the TransplantLines biobank and cohort study. Nephrol Dial Transplant 2023;38:1719–28. 10.1093/ndt/gfad013 36662046 PMC10310504

[6] Ziengs AL, Buunk AM, Van Sonderen L et al. Long-term cognitive impairments in kidney transplant recipients: impact on participation and quality of life. Nephrol Dial Transplant 2022;38:491–8. 10.1093/ndt/gfac035 PMC9923696 35175356

[7] Karakizlis H, Doerr JM, Becker A et al. Neuropsychological Assessment of Cognitive impairment in Kidney Transplantation (NAsKiT) and its related risk factors: a study protocol. J Nephrol 2022;35:1933–41. 10.1007/s40620-022-01376-z 35763254 PMC9458686

[8] Posselt J, Harbeck B, Rahvar A‐H et al. Improved cognitive function after kidney transplantation compared to hemodialysis. Ther Apher Dial 2021;25:931–8. 10.1111/1744-9987.13625 33497026

[9] Van Sandwijk MS, Ten Berge IJM, Caan MWA et al. Cognitive improvement after kidney transplantation is associated with structural and functional changes on MRI. Transplant Direct 2020;6:e531. 10.1097/TXD.0000000000000976 32195322 PMC7056275

[10] Murray AM, Tupper DE, Knopman DS et al. Cognitive impairment in hemodialysis patients is common. Neurology 2006;67:216–23. 10.1212/01.wnl.0000225182.15532.40 16864811

[11] Kalirao P, Pederson S, Foley RN et al. Cognitive impairment in peritoneal dialysis patients. Am J Kidney Dis 2011;57:612–20. 10.1053/j.ajkd.2010.11.026 21295896 PMC3121243

[12] Kurella Tamura M, Wadley V, Yaffe K et al. Kidney function and cognitive impairment in US adults: the reasons for geographic and racial differences in Stroke (REGARDS) study. Am J Kidney Dis 2008;52:227–34. 10.1053/j.ajkd.2008.05.004 18585836 PMC2593146

[13] Elewa U, Sanchez-Niño MD, Martin-Cleary C et al. Cardiovascular risk biomarkers in CKD: the inflammation link and the road less traveled. Int Urol Nephrol 2012;44:1731–44. 10.1007/s11255-012-0271-4 22965378

[14] Kurella Tamura M, Chertow GM, Depner TA et al. Metabolic profiling of impaired cognitive function in patients receiving dialysis. J Am Soc Nephrol 2016;27:3780–7. 10.1681/ASN.2016010039 27444566 PMC5118491

[15] Tryc AB, Alwan G, Bokemeyer M et al. Cerebral metabolic alterations and cognitive dysfunction in chronic kidney disease. Nephrol Dial Transplant 2011;26:2635–41. 10.1093/ndt/gfq729 21216887

[16] EAU , *Guidelines. Edn. presented at the EAU Annual Congress Milan 2023*. 2023;

[17] Chadban SJ, Ahn C, Axelrod DA et al. KDIGO Clinical Practice Guideline on the evaluation and management of candidates for kidney transplantation. Transplantation 2020;104:S11–s103. 10.1097/TP.0000000000003136 32301874

[18] Knoll G . Canadian Society of Transplantation Consensus Guidelines on eligibility for kidney transplantation. Can Med Assoc J 2005;173:1181–4. 10.1503/cmaj.051291 16275969 PMC1277045

[19] Kasiske BL, Ravenscraft M, Ramos EL et al. The evaluation of living renal transplant donors: clinical practice guidelines. Ad hoc Clinical Practice Guidelines Subcommittee of the Patient Care and Education Committee of the American Society of Transplant Physicians. J Am Soc Nephrol 1996;7:2288–313. 10.1681/ASN.V7112288 8959619

[20] Wyld MLR, Wyburn KR, Chadban SJ. Global perspective on kidney transplantation: Australia. Kidney360 2021;2:1641–4. 10.34067/KID.0003692021 35372973 PMC8785793

[21] Campbell S, Pilmore H, Gracey D et al. KHA-CARI guideline: recipient assessment for transplantation. Nephrology (Carlton) 2013;18:455–62. 10.1111/nep.12068 23581832

[22] Abramowicz D, Cochat P, Claas FHJ et al. European Renal Best Practice guideline on kidney donor and recipient evaluation and perioperative care. Nephrol Dial Transplant 2015;30:1790–7. 10.1093/ndt/gfu216 25007790

[23] Beauchamp TL, Childress JF. Principles of Biomedical Ethics. 8th edn. 2019; New York: Oxford University Press. pages cm.

[24] Davison SN, Holley JL. Ethical issues in the care of vulnerable chronic kidney disease patients: the elderly, cognitively impaired, and those from different cultural backgrounds. Adv Chronic Kidney Dis 2008;15:177–85. 10.1053/j.ackd.2008.01.004 18334244

[25] Singh-Manoux A, Oumarou-Ibrahim A, Machado-Fragua MD et al. Association between kidney function and incidence of dementia: 10-year follow-up of the Whitehall II cohort study. Age Ageing 2022;51:1–7. 10.1093/ageing/afab259 PMC8782607 35061870

[26] Kelly DM, Rothwell PM. Disentangling the relationship between chronic kidney disease and cognitive disorders. Front Neurol 2022;13:830064. 10.3389/fneur.2022.830064 35280286 PMC8914950

[27] Gupta A, Mahnken JD, Bernal J et al. Changes in cognitive function after kidney transplantation: a longitudinal cohort study. Am J Kidney Dis 2024; 10.1053/j.ajkd.2023.12.022 PMC11443557 38423160

[28] Faden RR, Beauchamp TL, King NMP. A History and Theory of Informed Consent. 1986; New York: Oxford University Press, 392.

[29] Petrini C . Informed consent in experimentation involving mentally impaired persons: ethical issues. Ann Ist Super Sanita 2010;46:411–21. 21169673 10.4415/ANN_10_04_09

[30] APA . Guidelines for assessing the decision-making capacities of potential research subjects with cognitive impairment. Am J Psychiatry 1998;155:1649–50. 9812160

[31] Shrestha P, Van Pilsum Rasmussen SE, King EA et al. Defining the ethical considerations surrounding kidney transplantation for frail and cognitively impaired patients: a Delphi study of geriatric transplant experts. BMC Geriatr 2022;22:566. 10.1186/s12877-022-03209-x 35804289 PMC9264705

